# A New Thinking: Deciphering the Aberrance and Clinical Implication of IGF Axis Regulation Pattern in Clear Cell Renal Cell Carcinoma

**DOI:** 10.3389/fimmu.2022.935595

**Published:** 2022-07-22

**Authors:** Aimin Jiang, Xiaofeng Wu, Desheng Wang, Anbang Wang, Kai Dong, Bing Liu, Le Qu, Peng Luo, Jian Wang, Qiang Tong, Linhui Wang

**Affiliations:** ^1^ Department of Urology, Changhai Hospital, Naval Medical University, (Second Military Medical University), Shanghai, China; ^2^ Department of Urology, The Second People’s Hospital of BengBu, Bengbu, China; ^3^ Department of Urology, Changzheng Hospital, Naval Medical University, (Second Military Medical University), Shanghai, China; ^4^ Department of Urology, The Third Affiliated Hospital, Naval Medical University (Second Military Medical University), Shanghai, China; ^5^ Department of Urology, Affiliated Jinling Hospital, Medical School of Nanjing University, Nanjing, China; ^6^ Department of Oncology, Zhujiang Hospital, Southern Medical University, Guangzhou, China; ^7^ Department of Urology, The Second People’s Hospital of Nantong, Nantong, China; ^8^ Department of Urology, No. 905 Hospital of PLA (Chinese People’s Liberation Army) Navy, Shanghai, China

**Keywords:** clear cell renal cell carcinoma, IGF, molecular subtypes, tumor immunity, multi-omics, SHC1

## Abstract

**Rationale:**

The recent research found that IGF regulator genes played a pivotal role in multiple biological processes, which may be developed for cancer treatment. However, the characteristics and implication of IGF regulators in cancers, especially in clear cell renal cell carcinoma (ccRCC), remain elusive.

**Methods:**

We systematically analyzed the expression, prognostic valuation, genome variation, and functional implication at pan-cancer level from The Cancer Genome Atlas. According to expression levels of IGF regulator genes, ccRCC could be divided into three different subtypes *via* unsupervised cluster algorithm: IGF pattern cancer type1 (IPCS1), type2 (IPCS2), and type3 (IPCS3). The immune microenvironment, immunotherapy response, metabolic pattern, and tumor progression signature among the three subgroups were investigated. The clinical characteristics, genomic mutations, and potential drug sensitivity were further analyzed. IGF pattern–related risk model was constructed to predict RCC patients’ outcome. Finally, SHC1, a potential IGF axis target, was comprehensively investigated in ccRCC.

**Results:**

We found that IGF regulator genes were specifically upregulated in various cancer tissues, which were correlated with copy number variations and dysregulated pathways. IPCS1, IPCS2, and IPCS3 exhibited different clinical profiles and biological characteristics in ccRCC. IPCS3 subtype indicated a higher clinical stage and a worse survival. IPSC3 ccRCC displayed activated metabolic signatures to fuel the cancer progression. IPCS3 subgroup holds a higher tumor mutation burden and lower immune activities, which resulted in a low ICI therapy response and tumor immunity dysfunction state. The genome copy numbers of IPCS2/3, including arm gain and arm loss, were significantly higher than IPCS1. Besides, the drug sensitivity profiles were different among the three subgroups. The prognostic risk model based on subtype’s biomarker exerted a promising performance both in training and validation cohorts. Finally, upregulated expression of SHC1 partly induced poorer immunotherapy response and shorter survival of ccRCC patients.

**Conclusion:**

Targeting IGF regulators may be functioned as a treatment approach among multi-cancers. IGF regulator–related signature could reshape the tumor immune microenvironment *via* activating multi-step immune programs. The inhibition of SHC1 may enhance the efficacy of immunotherapy, and SHC1 could be a suitable target for ccRCC therapy.

## Introduction

Renal cell carcinoma (RCC) is one of the most common malignant cancers of the urinary system. It is estimated that there are more than 431,000 new cases of RCC in the whole world, of which approximately 180,000 deaths are documented (1). Clear cell RCC (ccRCC) is the most common type of RCC, consisting of approximately 75% cases of RCC. Early-stage RCC often indicates no obvious symptoms. Approximately 30% of RCC patients have metastasized at the time of initial diagnosis, and nearly one-third of patients would eventually suffer recurrence and metastasis after resection of primary tumor (2, 3). Since metastatic renal cancer is not sensitive to radiotherapy and chemotherapy, there are limited drugs for clinical treatment of RCC. The prognosis of metastatic RCC is extremely poor with the 5-year survival rate of less than 5% (4). The targeted therapy and immunotherapy brought some hope for RCC patients; however, only some patients gain drug responsiveness, and most of them would develop treatment resistance. Derosa et al. reported that gut bacteria composition may affect drug responsiveness of immunotherapy in RCC. Homeostasis of the gut microbiota enhanced the efficacy of immunotherapy (5). In clinical practice, TNM classification and Fuhrman grade are the most widely used models for renal cancer risk classification and prognosis prediction. Due to the existence of tumor heterogeneity, patients with the same stage and grade may have significantly different prognosis (6). The models based on molecular subtypes may be able to better predict the prognosis of RCC patients. Thus, it is compelling to develop new molecular subtypes to predict prognosis and guide treatment for ccRCC patients.

Insulin-like growth factor (IGF) axis regulator, as an important metabolic factor, has been found to be closely related to cancer onset and progression in a variety of human malignancies (7). Dysregulation of IGF axis strongly induced invasive clinical features of tumors. The activation of PI3K-AKT-MAPK pathways plays an important role in mediating IGF function in tumor, and it is often correlated to dysregulation of transcription factors. Epithelial-Mesenchymal transition (EMT), another oncogenic pathway, could be driven by IGF1-induced activation of the transcription factors. IGF-IR signaling is part of network of receptor tyrosine kinase (RTK)–initiated pathways. IGF-IR could cross-talk with several crucial RTKs, including EGFR, FGFR, PDGFR, HER-2, and androgen receptor. In addition, IGF axis could enhance immunosuppression to promote cancer expansion. For instance, IGF-IR activation was correlated with macrophage polarization and pro-tumorigenic M2 phenotype (8). Correspondingly, dual IGF1R/IR inhibitors have been developed and indicated efficacy in several cancers (9). The combination of immunotherapy and IGF-targeting drugs may improve the efficacy of immunotherapy in solid cancers. However, the function of IGF axis regulator in the tumorigenesis and progression of ccRCC remains largely unknown.

In this study, we performed an IGF axis–related gene signature study by pan-cancer analysis and stratified analysis of ccRCC patients by integrating multi-omics data, including molecular features, biological function, immune infiltration, and drug sensitivity analysis. A reliable risk model, named RCIP4, was also constructed to verify the crucial role of IGF regulators in the prognosis prediction of ccRCC patients. In addition, we identified a promising IGF regulator target SHC1, which could function as a new therapy target for ccRCC.

## Materials and Method

### Data Collection and Processing

The workflow of this study was depicted in **Figure S1**. Pan-cancer normalized expression profiling data, DNA methylation data, tumor mutation burden (TMB), microsatellite instability (MSI), copy number variation (CNV) and somatic mutation data, and clinical characteristics were downloaded from UCSC XENA dataset (http://xena.ucsc.edu/), including the ccRCC-KIRC cohort (10). The Cancer Genome Atlas contains more than 10,000 patient samples for 33 cancers, which facilitated the pan-cancer study. The external ccRCC cohort, E-MTAB-3267, which included expression profile and prognostic information, was downloaded from ArayExpress database (https://www.ebi.ac.uk/arrayexpress/), and different stage single-cell sequence data of ccRCC patients was collected from GEO (ID PRJNA705464; https://www.ncbi.nlm.nih.gov/geo/). This study also facilitated with several public cancer databases, including UALCAN (http://ualcan.path.uab.edu/index.html), TIMER (https://cistrome.shinyapps.io/timer/), Tumor Immune Dysfunction and Exclusion (TIDE; http://tide.dfci.harvard.edu/), and MEXPRESS (https://mexpress.be/). Ethical Review Committee approval and informed consents were not required for datasets downloaded from public datasets. Patients without prognostic information or expression profiles were excluded from the study.

### Identification of Distinct IGF Axis Subgroups in ccRCC

We collected all IGF regulator genes according to prior researches and reviews (**Table S1**). R package “corrplot” was used to assess the correlation among these RNA modification regulators *via* Spearman’s and Pearson’s rank correlation algorithm. Consensus clustering was performed according to the expression matrix of RNA modification regulators *via* R package “ConsensusClusterPlus” (detailed parameters turn to reps = 100, pItem = 0.8, clusterAlg = “km”, and distance = “euclidean”). The subtype number k = 2 was determined as the best classification number, and the two subgroups were named as RMCS1 and RMCS2 subtypes, respectively.

### Enrichment Analysis Between Subgroups

R package “DEseq2” was used to identify differentially expressed genes (DEGs) between subgroups, and thresholds were set at adjusted p < 0.01 and the abstract log fold change >2. After calculating the DEGs, R package “ClusterProfiler” was used to perform Gene Ontology (GO), Kyoto Encyclopedia of Genes and Genomes (KEGG) pathways, gene set enrichment analysis (GSEA), and gene set variation analysis (GSVA), aiming to explain the biological function and molecular mechanism between RMCS1 and RMCS2. All gmt files used for enrichment analysis were downloaded from the MSigDB (11) (https://www.gsea-msigdb.org/gsea/index.jsp) and ConsensusPathDB (12) (http://cpdb.molgen.mpg.de/) databases.

### Differences in Immune Infiltration Signatures and Therapy Response

We utilized multiple immune cell infiltration algorithms including TIMER, CIBERSORT, QUANTISEQ, MCPCOUNTER, XCELL, and EPIC to calculate cellular components or immune cell enrichment scores in ccRCC tissues to compare tumor microenvironment (TME) components between subgroups. In addition, single-sample GSEA (ssGSVA) was introduced to further validate differences of immune cell infiltration between RMCS1 and RMCS2 (13–16). R package “ESTIMATE” was used to evaluate the stromal and immune scores based on ccRCC tissues’ expression profiling. The TIDE (http://tide.dfci.harvard.edu/) algorithm (17) was used to compare immunotherapy responses between subgroups.

### Mutation Spectrum Characteristics Among Subpopulations

Somatic data were analyzed and visualized *via* R package “maftools“ to compare mutational patterns among subgroups (18). With the aid of correlation functions in R package “maftools”, the tumor mutation panorama, base transitions and transversions, single-nucleotide variants, mutation rates of alleles, copy number mutations, mutually exclusive or coexisting mutations, and gene mutation survival rates were calculated as previously reported (19). Through the transformation analysis function module, the drug and gene interactions and the differences in oncogenic signaling pathways of different subsets were also analyzed. Analysis of recurrent extensive and focal somatic copy number alterations (SCNA) was performed by the GISTIC 2.0 (20) (https://cloud.genepattern.org/gp/pages/index.jsf) algorithm based on Euclidean distance of threshold copy number (21).

### Drug Susceptibility Prediction

Each patient was assessed for their susceptibility to molecular drugs using the Genomics of Cancer Drug Sensitivity (GDSC; https://www.cancerrxgene.org/) database. R package “pRRophetic” was used to estimate the half-maximal inhibitory concentration (IC_50_) and cross-validated. In addition, the CellMiner (22) (https://discover.nci.nih.gov/cellminer/home.do) and CCLE (23) (https://sites.broadinstitute.org/ccle) databases were also introduced to compare the different sensitivities between ccRCC cell lines. Spearman’s correlation coefficient was used to identify whether gene expression was associated with drug sensitivity. A positive correlation means that high expression of the gene indicates resistance to the drug, and low expression of the gene indicates sensitivity to the drug.

### Construction of Risk Prediction Model Related to IGF Regulator Genes

Firstly, using subgroup-related biomarkers and overall prognostic information from the TCGA-KIRC cohort, univariate Cox regression analysis was performed to select survival-related signatures. Then, the random survival forest variable hunting (RSFVH) algorithm was further performed to select crucial signatures. Finally, a risk scoring model was constructed using the best combination of prognostic genes to screen. The JAPAN-ccRCC cohort was used to validate our risk scoring model, and patients in both datasets were divided into high- and low-risk groups based on median risk scores.

### Statistical Analysis

All data processing, statistical analysis, and plotting were performed *via* R software (version 4.0.4). Differences between subgroups were compared by Kruskal–Wallis test and Wilcoxon test. Differences in clinical characteristics and inhibitor response between subgroups were compared by the chi-square test. Differences in prognosis, including overall survival (OS) and progression-free survival (PFS), were compared by the Kaplan–Meier method and Log-rank test. Hazard ratio (HR) differences were calculated by univariate Cox regression and multiple Cox regression analyses. Two-way p-values were taken and P < 0.05 was considered statistically significant. P-value was adjusted by the Benjamini–Hochberg (BH) multiple testing method.

## Results

### Dysregulation and Mutation of IGF Regulators in Cancers and Their Correlations With Patient Outcomes

**Figure 1:**
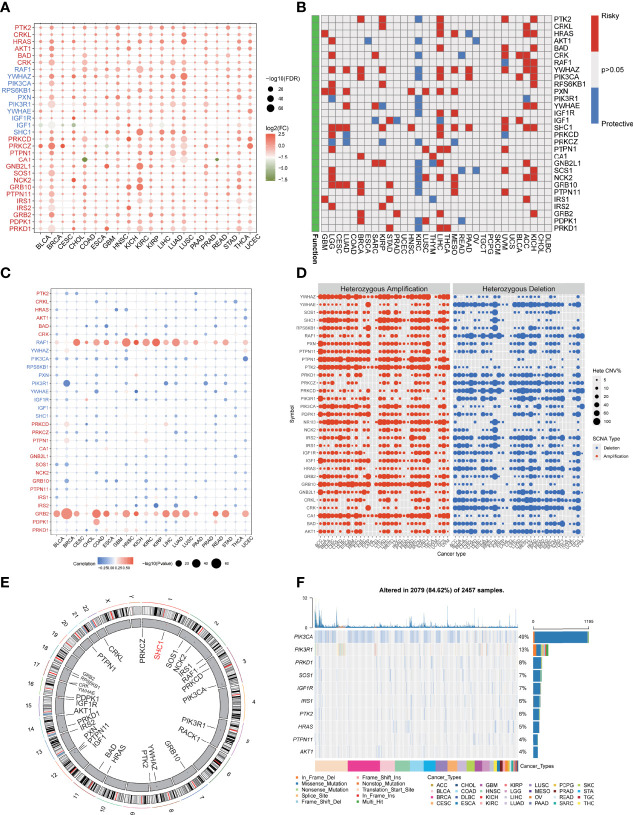
Dysregulation and genome alteration of IGF regulator genes in cancers. **(A)** The gene expressions of IGF regulator genes between multi cancers tissues and normal tissues. **(B)** The association between expression of IGF regulator genes and patient survival. **(C)** The correlation analysis of CNV with gene expression of IGF regulator genes. **(D)** Heterozygous amplification or deletion of IGF regulator genes in multi cancers. **(E)** The genome locations of IGF regulator genes on 23 chromosomes. **(F)** Mutation frequency and type of IGF regulator genes in multi cancers.

The study flow was shown in **Supplementary Figure 1**. To comprehensively explore the regulation pattern of IGF axis in multi cancers, we investigated the expression pattern of IGF regulators in pan-cancer. We found that the vast majority of IGF regulators were upregulated in various cancers, such as HRAS and YWHAZ in lung squamous cell carcinoma (LUSC), SHC1 in kidney cancer, PRKCZ in bladder cancer (BLCA) and in breast cancer (BRCA), and NCK2 in cholangiocarcinoma (CHOL) (**Figure 1A**). Survival analysis showed that most of these IGF regulators predicted poor prognosis of cancer patients (**Figure 1B**), indicating that the disturbed expression of IGF regulators played an important role in cancer development. Of interest, all of investigated regulators were protective factors for ccRCC.

To further understand the rationale of IGF regulators perturbation, we verified the CNV and single-nucleotide variation (SNV) of them in pan-cancer. As shown in **Figure 1C**, the significant correlations were observed between CNV and gene expression, especially RAF1 and GRB2 expressions were positively correlated with CNV. Heterozygous amplifications frequently presented in YWHAZ, PTPN1, PTK2, PIK3CA, and GRB10, whereas heterozygous deletions often appeared in YWHAE, RAF1, PRKCZ, PRKCD, and CRK (**Figure 1D**). Conversely, homozygous amplification and deletion rarely occurred (**Supplementary Figure 2A**). In ccRCC, we found CNV gain for PIK3CA, SHC1, and PTK2, whereas RAF1, PRKCD, and IRS1 were dominantly CNV loss (**Figure S2B**). The location of CNV alteration of IGF regulators on chromosomes was shown in **Figure 1E**. We also analyzed the SNV frequency of IGF regulator genes. The mutation frequency of IGF regulator genes was 84.62% (n = 2,079) in all tested samples (**Figure 1F**). The higher mutated genes included PIK3CA, PIK3R1, PRKD1, SOS1, and IGF1R. The SNV rate of CDKN2A was even close to 50%. Cancers with higher SNVs contained UCEC, BRCA, COAD, BLCA, and HNSC (**Supplementary Figure 2C**). Therefore, both transcriptional regulation and genome alteration together contributed to dysregulation of IGF regulators in multi-cancers.

### Identification of Three clusters by Clustering Analysis of IGF Regulators in ccRCC

**Figure 2:**
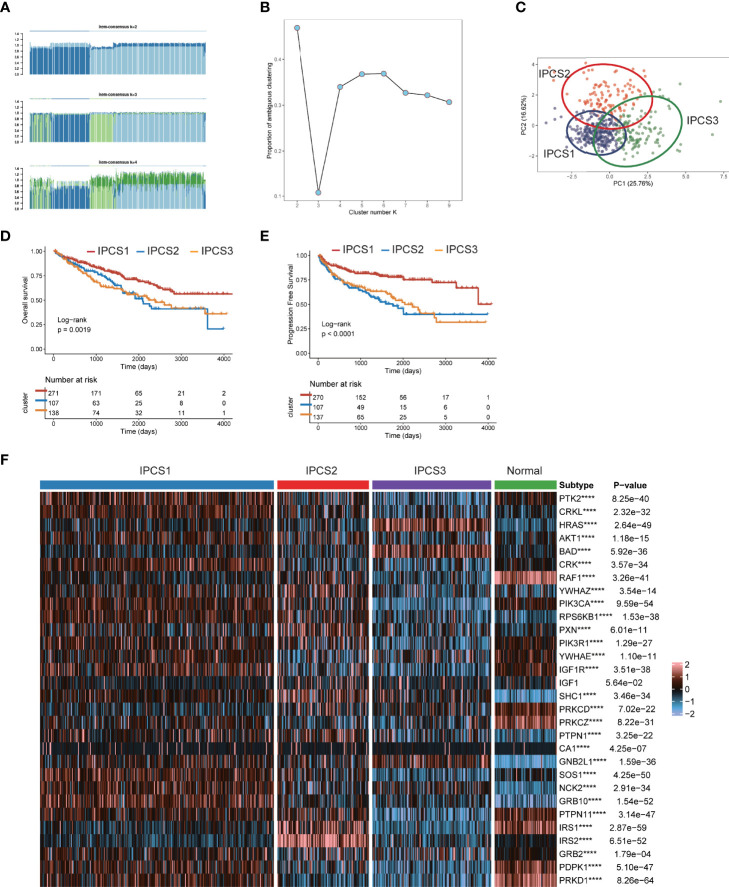
Screen of three clusters for IGF regulator genes in ccRCC. **(A)** Consensus matrix of samples in TCGA-ccRCC for k = 2, 3, or 4. **(B)** The cluster numbers are determined by the lowest proportion of ambiguous clustering. **(C)** The principal component plot is based on IGF regulator genes. **(D, E)** Survival analysis for overall survival (OS) and progression-free survival (PFS) among the three subtypes of TCGA-ccRCC. **(F)** The expression profiles of IGF regulator genes among the three subtypes and normal tissues. ****p < 0.0001.

As we suggested above, IGF regulators were protective factors for ccRCC, which was significantly different from other cancers. Thus, we further explored the characteristics of IGF regulator genes in ccRCC. According to expression levels of IGF regulator genes, TCGA-ccRCC samples were classified into different molecular subtypes using an unsupervised clustering method. We identified the optimal cluster number using the “Consensus Cluster Plus” R package and evaluated the subtyping reliability by a PAC algorithm. Consequently, TCGA ccRCC dataset was significantly divided into three clusters, namely, IGF pattern cancer type1 (IPCS1), type2 (IPCS2), and type3 (IPCS3) (**Figures 2A–C**). The patients’ clinical characteristics of three subtypes were compared and shown in **Supplementary Table 2**. Compared with IPCS1, IPCS2 and IPCS3 patients encompassed higher T stage and shorter OS and PFS (**Figure 2D**). In addition, we analyzed the expression of IGF regulator genes among three ccRCC subtypes and normal tissues. IPCS3 was regarded as desert of IGF axis, which expressed the lowest IGF regulator genes than other types (**Figure 2F**). Of interest, IRS1/2, SHC1, PXN, and YWHAZ expressed high levels in IPCS2. Conversely, HRAS and BAD showed a higher level expression in IPCS3. The desert of IGF regulator genes in IPCS3 leads to the inhibition of IGF axis, which trained IPCS3 to an aggressive clinical subgroup.

### Functional Enrichment Analysis of ccRCC Subtypes

Since the different clinical characteristics among each group, we next identified the different genes expression profiles among IPCS1, IPCS2, and IPCS3. The dysregulated genes were collected to perform functional enrichment analysis. Go analysis indicated that the upregulated genes were enriched in single fertilization and neuroactive ligand−receptor interaction for IPCS2 and ATP synthesis, oxidative phosphorylation, respiratory electron transport chain, and mitochondrial electron transport NADH to ubiquinone for IPCS3 (**Supplementary Figure 3A**). Collectively, the downregulated genes were dominant in positive regulation of urine volume, response to hepatocyte growth factor, and steroid hormone–mediated signaling pathway in IPCS1; cornification, keratinization, keratinocyte differentiation, and secretory granule localization in IPCS2; sperm egg recognition and negative regulation of execution phase of apoptosis in IPCS3 (**Supplementary Figure 3B**).

**Figure 3:**
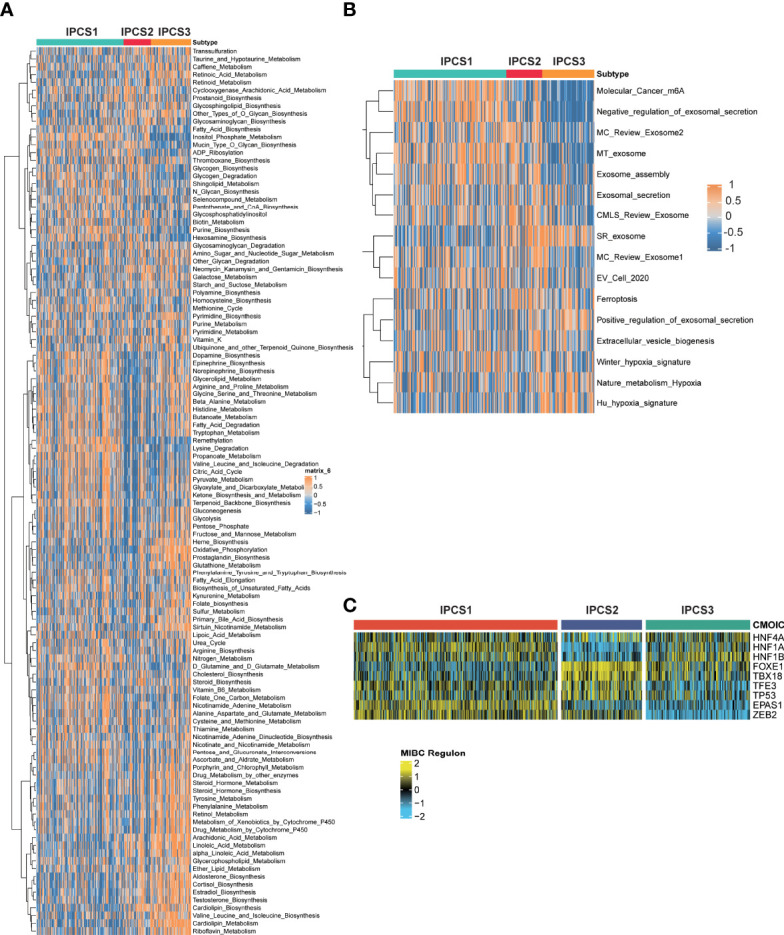
Functional enrichment analysis of ccRCC subtypes. The GSEA pathway analysis indicating heatmap of tumor metabolism–related pathways **(A)** and tumor microenvironment–related pathways **(B)** among the three subtypes. **(C)** Transcriptional factors that involved in the regulation among the three subtypes.

We then performed GSEA pathway analysis for metabolism-associated signatures. We observed partly repressive metabolic status in IPCS1, since some metabolic signatures including retinoic acid metabolism, taurine and hypotaurine metabolism, arachidonic acid metabolism, aldosterone biosynthesis, and valine leucine and isoleucine biosynthesis were significantly suppressed in IPCS1. On the contrary, most of these signatures were obviously activated in IPCS3, indicating its metabolic activation state (**Figure 3A**). Consistently, GSVA analysis showed that the hypoxia signature was activated in IPCS3 (**Figure 3B**). Hypoxic stress was an important feature of tumor metabolism and prompted resistance to immunotherapy in cancers (24, 25). Thus, targeted hypoxia pathway may re-sensitize IPCS3 to immunotherapy. Besides, m6A signature was significantly suppressed in IPCS3, revealing a potential link between m6A and IGF signaling (**Figure 3B**).

To further illustrate the transcriptome differences, iRegulon analysis was performed to analyzed transcriptional regulons for IGF axis using renal cancer–associated transcription factors. The difference of transcription factors rendered strong support to the biological classification, because the regulon activity was correlated with IGF-related subtypes (**Figure 3C**). We found that ZEB2 and EPAS1 exhibited the lowest activity in IPCS3, indicating the suppression of EMT and susceptibility of hypoxia in this subgroup. Zhu et al. reported that ZEB2 was considered as an immune-associated gene and associated with macrophage infiltration in cancer (26). Therefore, metabolic environment and transcriptional regulatory network together induced the formation of IGF-related subtypes, which functioned in regulating biological functions.

### Comparison of Immune Infiltration Landscape Among the Three Subtypes

**Figure 4:**
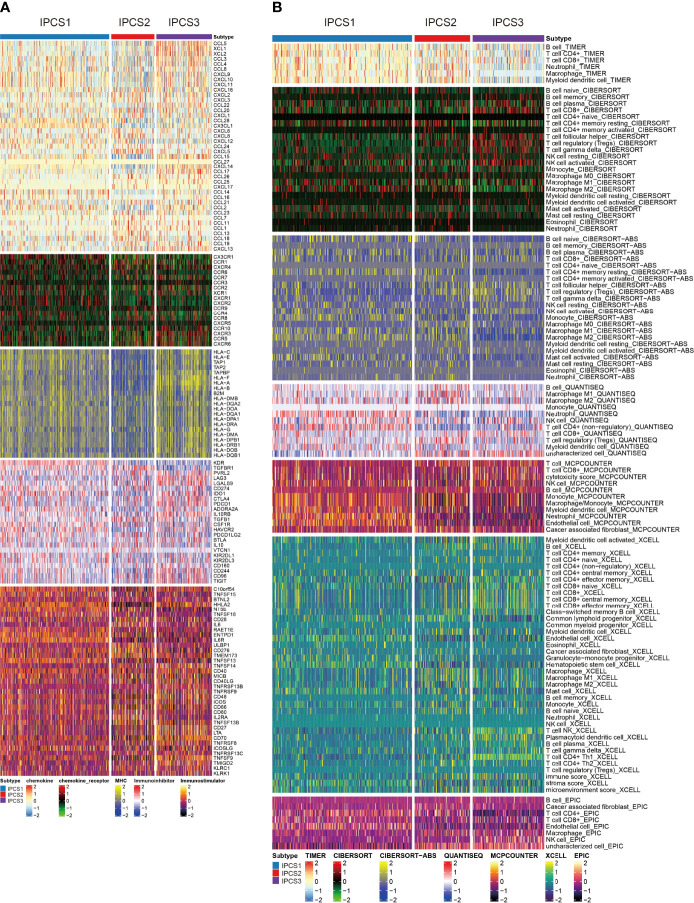
Investigations of immune profiling. **(A)** Heatmap of immune-related genes among IPCS1, IPCS2, and IPCS3. **(B)** Heatmap of tumor-related infiltrating immune cells based on TIMER, CIBERSORT, CIBERSORT-ABS, QUANTISEQ, MCPCOUNTER, XCELL, and EPIC algorithms among IPCS1, IPCS2, and IPCS3.

**Figure 5:**
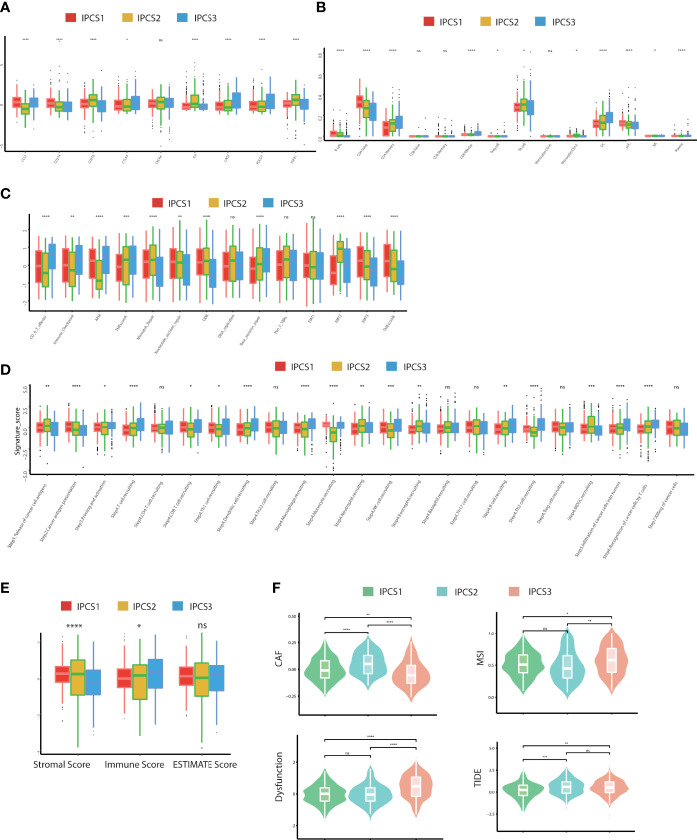
Landscapes of specific immune components and immune function scores. **(A-D)** The immune antigens, immune cells, immune pathways, and anti-cancer steps among the three subtypes. **(E)** ESTIMATE scores of two subtypes. **(F)** The immune function scores among the three subtypes. ns>0.05, p < 0.05, **p < 0.01, ***p < 0.001, ****p < 0.0001. ns, no significance.

Immunotherapy gradually becomes a dominant treatment way for ccRCC in recent years. To depict the immune characteristics, we analyzed immune infiltration environment across the subgroups using GSVA analysis. We found the difference of immune-related gene expression among three subgroups. IPCS3 subtype expressed a lower level of CXCL12, CCR1, CCR4, TGFBR1, CD274, IL6R, CD80, and IL2RA (**Figure 4A**). The compositions of TME infiltrating cell types were examined, and the results indicated that IPCS3 and IPCS2 displayed reduced immune cell infiltration compared to IPCS1 (**Figure 4B**). Thus, IPCS2/3 could be categorized as immune-desert subgroups, marked by the status of immune suppression. As discussed above, IPCS2/3 indicated matched poor prognosis comparing to IPCS1. We then observe an anti-cancer immune response, which was consist of a series of immune events. IPCS2/3 subgroups lacked plasmacytoid dendritic cells (DCs) and CD274 (PD-L1), which impeded immune cells to identify tumor cells (**Figures 5A,B**). IPCS3 subtype, lacking DNA damage repairing ability, was significantly correlated with CD8 T effector and immune checkpoint, which indicated the potential response to immunotherapy (**Figure 5C**). We observed lower activities of many steps in IPCS2/3, including release of cancer cell antigens (step1), cancer antigen presentation (step2), and CD8 T cell and T_H_1 cell recruiting (step4) (**Figure 5D**). Besides, the estimate algorithm suggested that IPCS3 subtype obtained the highest stromal score and the lowest immune score (**Figure 5E**). Immune function score analysis indicated that IPCS3 subtype gained higher MSI score, dysfunction score, and TIDE score (**Figure 5F**), which again verified the immunocompromised state of IPCS3. These results demonstrated that distinct immune functions were correlated with IGF axis signaling.

### Comparison of Tumor Somatic Mutations and CNVs Among the Three Subtypes

**Figure 6:**
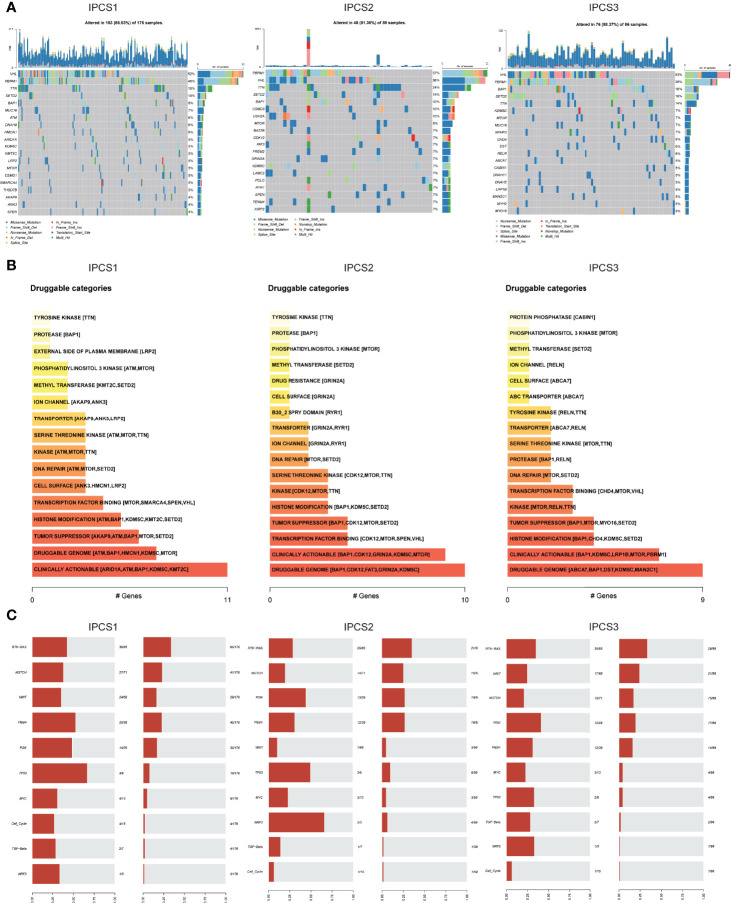
Profiles of somatic mutations and potential targets among the three subtypes. **(A)** Waterfall plot showing the mutation patterns of the top 20 most frequently mutated genes. **(B)** Potential druggable gene categories from mutation dataset among IPCS1, IPCS2, and IPCS3. **(C)** The fraction of pathways or samples of oncogenic signaling pathways among IPCS1, IPCS2, and IPCS3.

In addition to the influence of the immune infiltration on therapy, genome mutations partly accounted for drug effectiveness. IPCS3 subtype presented the highest mutation frequencies than IPCS1 and IPCS2 (**Figure 6A**). The waterfall charts depicted the top 20 mutant genes among the subtypes. IPCS3 subtype contained several higher frequently mutated genes, including BAP1, KDM5C, MUC16, CHD4, and DST. According to the mutation data, we used DGIdb database and drug interactions in maftools package to identify potential targets for each subgroup. Druggable genes in three different IGF axis patterns were classified into 16, 17, and 17 categories, respectively, including druggable genome, clinically actionable, histone modification, and tumor suppressor. (**Figure 6B**). We further investigated the somatic mutations in oncogenic pathways, such as RTK-RAS, NOTCH, WNT, Hippo, and PI3K using R package maftools. TP53 and Hippo were the most affected pathways in IPCS1, whereas NRF2 and TP53 were easily affected in IPCS2. In IPCS3, PI3K and RTK-RAS were the most altered oncogenic pathways (**Figure 6C**). The somatic interaction analysis indicated that co-mutation of VHL and RYR2 induced cell death in IPCS3, which provided a potential approach for subgroup treatment (**Supplementary Figure 4A**). Regarding mutations of IGF regulator genes, compared to IPCS1 and IPCS3, IPCS2 subtype reserved the highest mutation frequency, in which SOS1 was the gene with the highest mutation rate (**Supplementary Figure 4B**).

**Figure 7:**
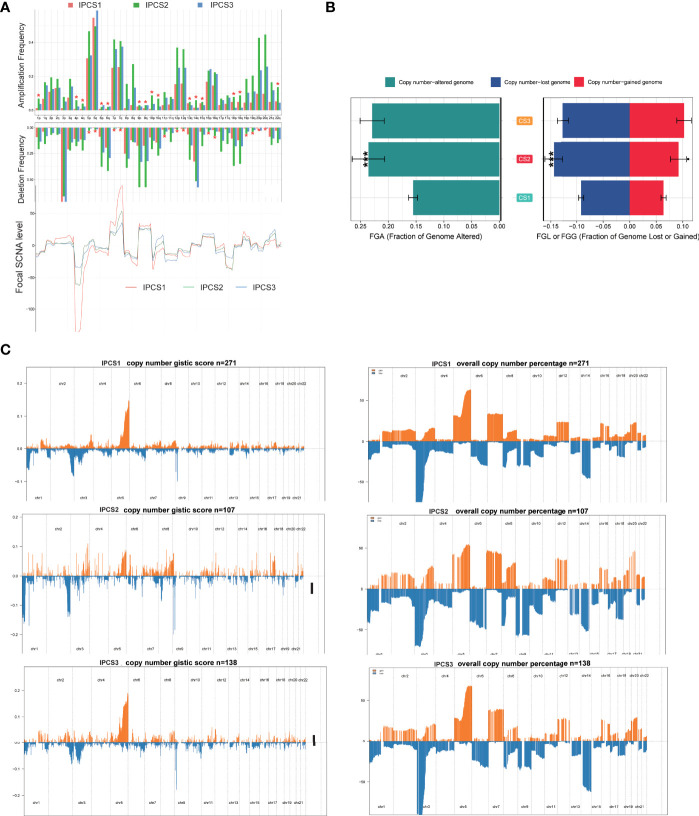
Landscapes of copy number variations among the three subtypes. **(A)** The amplification or deletion frequency in chromosomes among IPCS1, IPCS2 and IPCS3. **(B)** Barplot of genomic fractions altered among the three subtypes. (C) The GISTIC score and percentage of copy number profiles in ccRCC.

CNVs were also compared among the three subgroups. IPCS2 displayed the highest CNV rate, followed by IPCS3 and IPCS1 (**Figures 7A,B**). The amplification and deletion regions on chromosomes were identified and decoded using GISTIC 2.0 (**Supplementary Table 3**). The gain/loss percentage and GISTIC score indicated similar patterns (**Figures 7B, C**). The recurrent CNVs of IPCS3 included the amplification of 5q35.3 (CANX) and 5q15 (LIX1) and also the deletion of 9p21.3 (CDKN2A) and 9p23 (PTPRD). The specific CNVs of IPCS2 were the amplification of 1p31.3 (UBE2U) and 1q32.1 (ATP2B4) and also the deletion of 9p23 (PTPRD) and 9p21.3 (CDKN2A). Differences in genome mutations may lead to the formation of the three subtypes.

### Drug Sensitivity Analysis of Different IGF Subtypes

**Figure 8:**
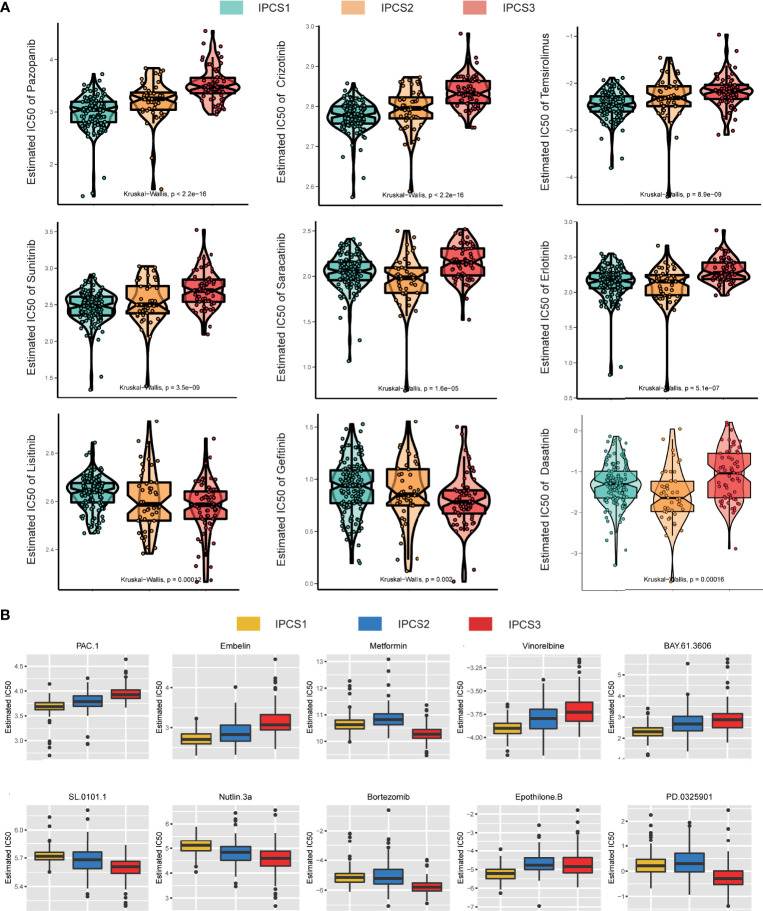
Drug sensitivity analysis of three subtypes. **(A)** Estimated IC_50_ of the indicated molecular-targeted drugs among the three subtypes. **(B)** Estimated IC_50_ of the potential molecular inhibitors in IPCS1, IPCS2, and IPCS3.

To explore potential drugs, the drug response data were collected from the GDSC database. We observed that most drugs were less effective against IPCS3 subgroup, which was consistent with the poor prognosis of IPCS3 (**Figure 8A**). IPCS3 was only predicted to be sensitive to linsitinib and gefitinib. In contrast, IPCS1 subtype was more sensitive to pazopanib, imatinib, crizotinib, temsirolimus, and sunitinib (**Figure 8A** and **Supplementary Figure 4C**), whereas IPCS2 subtype had better drug responsiveness to saracatinib, erlotinib, dasatinib, and afatinib (**Figure 8A** and **Supplementary Figure 4C**). We further analyzed drug responsiveness of different subtypes to 138 small molecular inhibitors (**Supplementary Table 4**). **Figure 8B** showed the top 10 drugs with a better drug response. IPCS1 subtype indicated sensitivity to PAC.1, embelin, vinorelbine, BAY.61.3606, and epothione B, whereas IPCS3 had a better response to metformin, SL.0101.1, nutlin 3a, bortezomib, and PD.0325901. Conceivably, metformin and SL.0101.1, the direct inhibitors of IGF and RTK-RAS pathway, may be utilized to develop effective targets for IPCS3 subtype.

### Verification of Robustness of the Classification Model Using External Dataset

**Figure 9:**
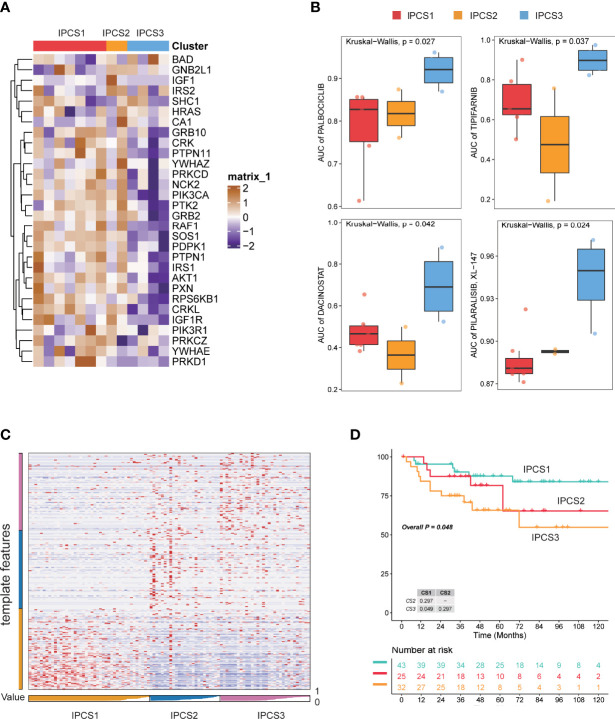
Verification of subtyping model in external dataset. **(A)** Heatmap of the expression profiles of IGF regulator–related genes among the three subtypes of GDSC renal cancer cells. **(B)** Drug sensitivity analysis in the form of normalized AUC using GDSC renal cancer cell database. **(C)** Heatmap of NTP in JAPAN-KIRC cohort using subtype-specific altered biomarkers identified from TCGA-ccRCC cohort. **(D)** Survival analysis of the three predicted subtypes of ccRCC in JAPAN-KIRC cohort.

To further confirm the reliability of the classification model, we conducted verification using the GDSC renal cancer database and JAPAN-KIRC cohort. The significant difference for ccRCC cell line was found among the three subtypes (**Figure 9A**). Consistent with TCGA cohort results, many IGF regulator genes were significantly downregulated in IPCS3 subgroup. We investigated the area under the curve (AUC) of drug sensitivity among the subgroups, and IPCS3 subtype had the highest AUC of all drugs. IPCS2 subtype was sensitive to palbociclib, tipifarnib, and dacinostat, whereas IPCS1 presented more sensitivity to pilaralisib (**Figure 9B**). Applying the nearest template prediction (NTP) algorithm, the subtype-specific hallmarks identified from TCGA-ccRCC could divide JAPAN-KIRC cohort into three distinct groups (**Figure 9C**). The IPCS3 group indicated poorer survival than the IPCS1 group, which was consistent with previous data (**Figure 9D**). These results verified the reliability and stabilization of the molecular subtyping model.

### Construction and Verification of a Four IGF-Related Genes Risk Model

**Figure 10:**
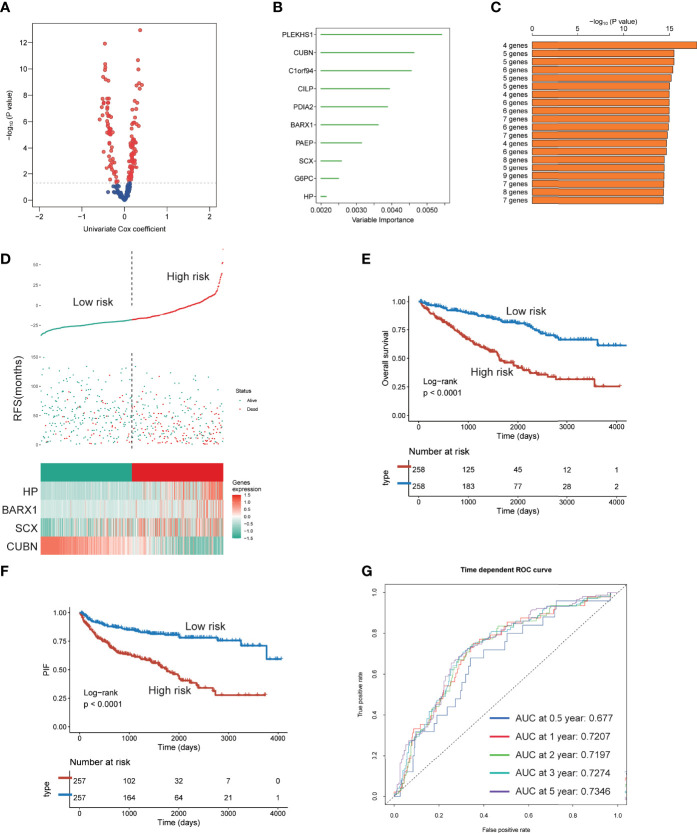
Establishment of a four IGF genes risk model. **(A)** Volcano plot indicating the significantly altered biomarkers among the three subtypes by univariable Cox regression analysis. **(B)** Random survival forest analysis screening 10 genes. **(C)** Based on various combination analyses, the top 20 signatures are listed according to p-values. **(D)** Risk score analysis in TCGA-ccRCC cohort. **(E-F)** Survival analysis for OS **(E)** and PFS **(F)** of the two risk groups in TCGA-ccRCC cohort. **(G)** The time-dependent ROC curves for the two risk groups in TCGA-ccRCC cohort.

Since the three subtypes displayed different clinicopathological signatures and molecular heterogeneity in biological function and immune profile, we identified genes affected patients’ OS using univariate Cox regression analysis and constructed an IGF-related genes risk model based on subtype’s signatures (**Figure 10A**). Subsequently, 10 most relevant genes were identified using the random forest supervised classification algorithm (**Figure 10B**). To construct the best risk assessment model, we used the Kaplan–Meier (KM) analysis and measure *p*-value of all risk models (**Figure 10C**). Consequently, the risk assessment model composed of four genes (CUBN, BARX1, SCX, and HP) was screened out and called RCIP4. The risk score of each patient was calculated as follows: RCIP4 = −5.048360* CUBN + 6.693172* BARX1 + 6.027303* SCX + 4.804082* HP. To validate the effectiveness of RCIP4, both TCGA-ccRCC and JAPAN-KIRC cohort patients were categorized as the high score and low score groups by a median cutoff score (**Figure 10 D** and **Supplementary Figure 5A**). Survival analysis indicated that patients with high score had poorer prognosis than with low score in the two cohorts (**Figures 10E,F** and **Supplementary Figure 5B**). The AUC curve was used to evaluate the sensitivity and specificity of the RCIP4 risk model. The AUC score for TCGA-ccRCC cohort was 0.677, 0.7207, 0.7197, 0.7274, and 0.7346 at 0.5, 1, 2, 3, and 5 years, respectively (**Figure 10G**). The predictive value of the RCIP4 model was also determined in JAPAN-KIRC cohort (**Supplementary Figure 5C**). The above results demonstrated that the RCIP4 model was effective and applicable for prognostic evaluation of ccRCC.

### The Core Role of SHC1 in ccRCC

**Figure 11:**
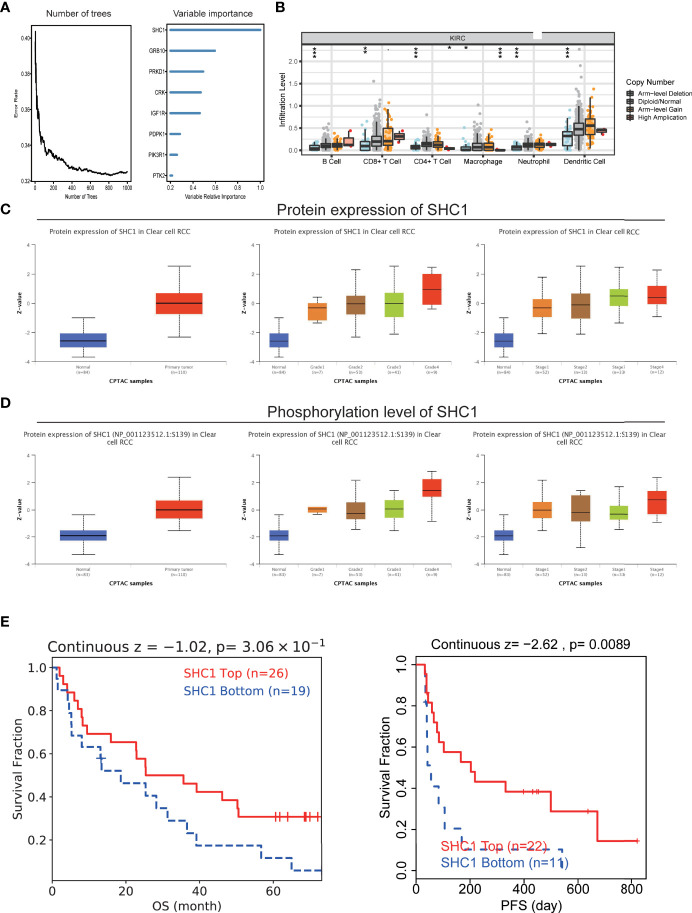
The hub role of SHC1 in ccRCC. **(A)** Number of trees showing the importance proportion of IGF regulator genes. **(B)** The association between SHC1 mutation and immune cell infiltration in ccRCC. **(C)** Protein levels of SHC1 in ccRCC samples, classified by tumor grade, and histological pathological stage using the UALCAN database. **(D)** Phosphorylation level of SHC1 in ccRCC samples, classified by tumor grade, and histological pathological stage. **(E)** The relationship between SHC1 expression and immunotherapy response.

Given the regulation of IGF-related genes in ccRCC, we analyzed which gene shared the most proportion of importance. We observed that SHC1 may play the core role in IGF axis signaling (**Figure 11A**). SHC1 mutation was correlated with immune infiltration levels of B cell, CD4^+^ T cell, CD8^+^ T cell, macrophage, neutrophil, and DC (**Figure 11B**). We found that SHC1 was associated with abundant signaling pathways in pan-cancer, such as TNFA-NFKB, inflammatory response, IL6-JAK-STAT3, hypoxia, EMT, apical junction, and angiogenesis (**Supplementary Figure 5D**). As previously discussed, SHC1 protein expression was also upregulated in renal cancer, and the magnitude of regulation increased with the grades and stages in CPTAC-ccRCC samples (**Figure 11C**). We also evaluated the phosphorylation level of SHC1 in cancer and normal tissues, which indicated an increased level of phosphorylated SHC1 in ccRCC. Besides, the phosphorylation level of SHC1 increased with the progress of the tumor stage and grade in CPTAC-ccRCC samples (**Figure 11D**). We also compared the immunotherapy response in high or low SHC1 expression groups. The high SHC1 expression group showed poorer immunotherapy response and shorter survival than the low SHC1 group (**Figure 11E**). Therefore, it makes sense to prompt immunotherapy response by inhibiting SHC1 expression.

## Discussion

ccRCC is characterized by tumor heterogeneity and immunogenicity (27). It is compelling to predict patients’ survival and enhance immunotherapy response. There was emerging evidence indicated that IGF axis signaling was involved in tumor onset, progression, and drug resistance in several cancers. However, an IGF axis study of ccRCC was not as abundant as that of other cancers. Most of related studies focused on signal regulatory molecule, and the global features induced by IGF axis signaling have not been fully understood. Nonetheless, IGF-based molecular subtyping in tumor pools has hardly been explored.

In this study, we analyzed IGF regulator genes in more than 10,000 samples of 33 cancers by mining multi-omics data. We observed that IGF regulator genes were significantly upregulated in various cancer tissues compared with adjacent normal tissues, which were related to genome mutation and epigenetic modification. Based on the expression of IGF regulator genes, ccRCC patients could be grouped into three distinct IGF regulation subtypes (IPCS1, IPCS2, and IPCS3). Most of those IGF regulator genes indicated poor survival in cancers, except ccRCC. IPCS3 subtype retains higher TMB, activated metabolic signatures, and immune suppression status, along with poor survival than other subtypes. Moreover, the prognostic risk model constructed based on subgroups hallmarks obtained promising results both in training and validation cohorts. Finally, SHC1, as the hub gene of IGF axis, could be utilized as a reliable target for ccRCC.

Immunotherapy, based on immune checkpoint inhibitor, has become the first-line treatment choice for ccRCC. However, only some patients respond well to immunotherapy, and drug resistance is inevitable. A possible solution is combination therapy, in which IGF regulator target may collaborate with immunotherapy. The compelling evidence showed that IGF axis signaling enhanced immunosuppressive response in TME. We found that IPCS3 subtype expressed lower level of immune components and immune cell infiltration, marked by the status of immune suppression. Consistent with our finding, a recent study suggested that IGF-I negatively regulated DC activation, impeded antigen-presenting process, and stimulated the proliferation of immunosuppressive regulatory T cells (T_reg_) (28, 29). Antigen presentation is a critical step for immune cells to eliminate tumors. We observed lower activities of many pivotal steps in IPCS2/3, such as antigen presentation and CD8 T-cell recruiting. Huang et al. found that IGFs inhibited DC-mediated anti-tumor immunity through regulating ERK1/2 phosphorylation and p38 dephosphorylation, and IGF inhibitor NVP-AEW541 restored DC-mediated antigen presentation process (30). Moreover, the inhibitor NT157 that targeted both the IGF-IR and STAT3 suppressed the expression of pro-tumorigenic cytokines, chemokines, and growth factors, including IL-6, CCL2, and TGF (31). Wu et al. found that IGF1 receptor inhibitor amplified the effects of cancer drugs by autophagy and immune-dependent mechanisms (32). IGF axis as a contributor for pro-tumorigenic TME could potentiate escape mechanisms from immune-mediated tumor cell eradication. Our results demonstrated that IPCS3, as a drug refractory subtype, was significantly correlated with CD8 T effector and immune checkpoint, which indicated the potential response to immunotherapy. Combined inhibition of IGF and immune checkpoints may reshape ccRCC microenvironment and promote drug effects *via* restoring immune homeostasis.

In addition to immune infiltration, IGF regulator was involved in many signaling pathways dysregulation in cancers. Our results found that IPCS1 was associated with normal renal function, whereas IPCS3 subtype was correlated with ATP synthesis, oxidative phosphorylation, respiratory electron transport chain, and mitochondrial electron transport NADH to ubiquinone. These activated energy supply pathways fuel cancer cells to expansion. Most of metabolism-associated signatures were obviously activated in IPCS3, indicating its metabolic activation state. Oxidative phosphorylation happens in mitochondria to generate ATP for cell energy. Oxidative phosphorylation was flexible and different in distinct tumors, such as leukemias and pancreatic ductal adenocarcinoma (33). Obesity is a multifaceted disease associated with an increased risk of cancer, including RCC. Obesity itself is a metabolic disease in which the IGF signaling axis plays a key role. There is growing evidence that both obesity and diabetes are potential literature factors for kidney cancer. A multi-institutional analysis of 2,597 patients with ccRCC showed that diabetes was an independent prognostic factor in terms of recurrence-free and OS (34). Our team also found that the thermogenic activity of adjacent adipocytes fueled the progression of ccRCC and compromised drug efficacy of tyrosine kinase inhibitors (35). IPCS3 was activated for the hypoxia signature, which shaped aggressiveness and metastatic potential and treatment resistance of solid cancers. HIF-1α cross-talk with HIF-2α maintains a balanced state of tumor cells’ demand for hypoxic factors (36). Hu et al. reported that hypoxia-induced lncHILAR promoted cancer cells metastasis *via* ceRNA for the miR-613/206/1-1-3p/Jagged-1/Notch/CXCR4 signaling pathway in RCC (37). Scharping et al. found that metformin, a widely used diabetes drug, inhibited oxygen consumption in tumor cells, resulting in decreased intratumoral hypoxia (24). The combination of metformin with PD-1 blockade leads to improved intratumoral T-cell function and tumor clearance.

ccRCC is one of the tumors with a high tumor mutational burden. The biological function of IGF regulator genes was correlated with genome mutations. IPCS3 subtype presented the highest mutation frequencies than IPCS1 and IPCS2. IPCS3 subtype reserved several higher frequently mutated genes, including BAP1, KDM5C, and CHD4. BAP1 was a critical tumor suppressor gene in ccRCC, prompting tumor development when mutated in the somatically (38). Loss of BAP1 promoted mesenchymal–epithelial transition process in renal cancer (39). The mutation of KDM5C enhanced ccRCC tumorigenicity by reshaping glycogen metabolism and inhibiting ferroptosis (40). Wang et al. reported that CHD4 promoted breast cancer progression through interaction with HIF (41). PI3K and RTK-RAS were the most altered oncogenic pathways in IPCS3. Both of these pathways were classical oncogene mutation pathways and played an important role in renal cancer progression. For CNVs, IPCS2 displayed the highest CNV rate, followed by IPCS3 and IPCS1. Fernandes et al. reported that most significant copy number alterations of ccRCC were loss of 3p (87.3%), 14q (35.8%), and 6q (29.3%) and also gains of 5q (59.7%), 7p (29.3%), and 16q (20.6%). Genes mapping to CNA significant regions included SETD2, BAP1, FLT4, PTEN, FGFR4, and NSD1 (42), which was consistent with our findings. Therefore, IGF regulators were involved in tumor heterogeneity through cross-talk with genome mutations.

As previously reported, IGF regulators affected the efficacy of anti-tumor drugs. ccRCC patients in different subtypes displayed distinct sensitivities to drugs, which could provide certain clinical guidance for treatment. We identified several potential molecular inhibitors for drug refractory IPCS3 subtype. Metformin and SL.0101.1, the direct inhibitors of IGF and RTK-RAS pathway, achieved good efficacy for the treatment of IPCS3. Notably, we constructed a risk model (RCIP4) to predict RCC patients’ survival. The four most relevant genes were included, i.e., CUBN, BARX1, SCX, and HP. Niinivirta et al. found that the combined expression of PKLR and CUBN could more accurately predict drug the responsiveness of sunitinib and sorafenib (43). Sun et al. indicated that transcription factor BARX1 contributed to the progression of ccRCC *via* promoting proliferation and epithelial–mesenchymal transition (44). A recent report found that SCX regulated Twist1 and Snai1 expression in the epithelial-to-mesenchymal transition (45). HP was regarding as a molecular biomarker in non–small cell lung cancer and glioblastoma (46, 47). We also verified the high accuracy of the RCIP4 risk model both in the TCGA-ccRCC and JAPAN-KIRC database. Of interest, we found that SHC1 may play the core role in IGF axis signaling. SHC1 was related to that activation of multiple oncogenic pathways. Recent reports indicated that SHC1 was a key driver of breast cancer initiation, and the SHC1 adaptor simultaneously balanced Stat1 and Stat3 activity to promote breast cancer immune suppression (48, 49). Lai et al. also found that DEPDC1B was a tumor promotor in development of bladder cancer through targeting SHC1 (50). The phosphorylation level of SHC1 increased with the progress of the tumor stage and grade in ccRCC samples. The higher SHC1 expression predicted poorer immunotherapy responsiveness, which suggested the rationale for enhancing the efficacy of immunotherapy by inhibiting SHC1.

Although our study characterized distinct IGF regulator signatures of ccRCC, some certain limitations are needed to be considered. Most of our findings were based on comprehensive bioinformatics analyses, and further experiment validations, such as the specific mechanism of IGF regulator function, were still necessary. Besides, the prognostic model may be affected by some confounding factors, such as race and area; thus, more independent datasets are needed to validate our risk model.

In summary, to our knowledge, this is the first study to comprehensively explore the function of IGF regulator genes in multi-cancers and to determine three molecular subtypes of ccRCC. Under certain conditions, inhibition of IGF regulators may become a suitable approach for cancer treatment. Such a signature could reshape the tumor immune microenvironment *via* impeding immune escape of cancer cells. Targeting SHC1 may have a synergistic effect with immunotherapy to eradicate tumor cells. Our study contributes to a better understanding of the relationship between IGFs and ccRCC, which can provide clinical guidance for ccRCC treatment.

## Data Availability Statement

The datasets presented in this study can be found in online repositories. The names of the “Pan-cancer profiling”, “TCGA-KIRC”, “E-MTAB-3267” and Single cell sequence data and accession number(s) can be found in the article/**Supplementary Material**.

## Author Contributions

AJ, XW, DW, and AW have contributed equally to this work. LW, QT, and JW conceptualized and designed this study. KD, BL, and LQ wrote the first draft of the manuscript. All authors contributed to the article and approved the submitted version.

## Funding

This work was supported by the National Natural Science Foundation of China (no. 81730073 and 81872074 to LW; no. 81772740 and 82173345 to LQ; no. 81902560 to AW), Foundation for Distinguished Youths of Jiangsu Province (no. BK20200006 to LQ).

## Conflict of Interest

The authors declare that the research was conducted in the absence of any commercial or financial relationships that could be construed as a potential conflict of interest.

## Publisher’s Note

All claims expressed in this article are solely those of the authors and do not necessarily represent those of their affiliated organizations, or those of the publisher, the editors and the reviewers. Any product that may be evaluated in this article, or claim that may be made by its manufacturer, is not guaranteed or endorsed by the publisher.

## Supplementary Material

Supplementary Figure 1 Workflow of this study.

Supplementary Figure 2 Genome mutation profile of IGF regulator genes in multi cancers. **(A)** The homozygous amplification/deletion of IGF regulator genes. **(B)** CNV of IGF regulators in TCGA-ccRCC dataset. CNV loss, blue; CNV gain, red; none CNV, green. **(C)** Mutation frequencies of IGF regulators genes in multi cancers.

Supplementary Figure 3 Functional enrichment analysis of ccRCC subtypes. **(A)** GO enrichment analysis of upregulated genes among the three subtypes. **(B)** GO enrichment analysis of downregulated genes among the three subtypes.

Supplementary Figure 4 The tumor somatic mutations and drug sensitivity assessment among groups. **(A)** The synthetic lethal mutations in IPCS1, IPCS2 and IPCS3. **(B)** Waterfall plot indicating the mutation patterns of IGF regulator genes in IPCS1, IPCS2 and IPCS3. **(C)** Estimated IC_50_ of the indicated molecular targeted drugs among the three subtypes.

Supplementary Figure 5 Verification of subtyping model in external dataset. **(A)** Risk score analysis of patients in JAPAN-KIRC cohort. **(B)** Survival analysis for OS of the two risk signatures in JAPAN-KIRC cohort. **(C)** The time-dependent ROC curves for the two risk signatures in JAPAN-KIRC cohort. **(D)** The correlation between SHC1 level and hallmark pathways in KIRC.

Supplementary Table 1 List of IGF regulator genes.

Supplementary Table 2 Clinicopathological features of different subtypes in ccRCC.

Supplementary Table 3 Recurrently amplification and deletion regions in the three subtypes calculated by GISTIC2.0.

Supplementary Table 4 List of discovered small-molecule drugs that could be used as potential drugs for the treatment of ccRCC.
