# Supplementary figures and images for: A New Thinking: Deciphering the Aberrance and Clinical Implication of IGF Axis Regulation Pattern in Clear Cell Renal Cell Carcinoma
Source: Front Immunol. 2022 Jul 22;13:935595. doi: 10.3389/fimmu.2022.935595 (PMC9355597; doi:10.3389/fimmu.2022.935595)

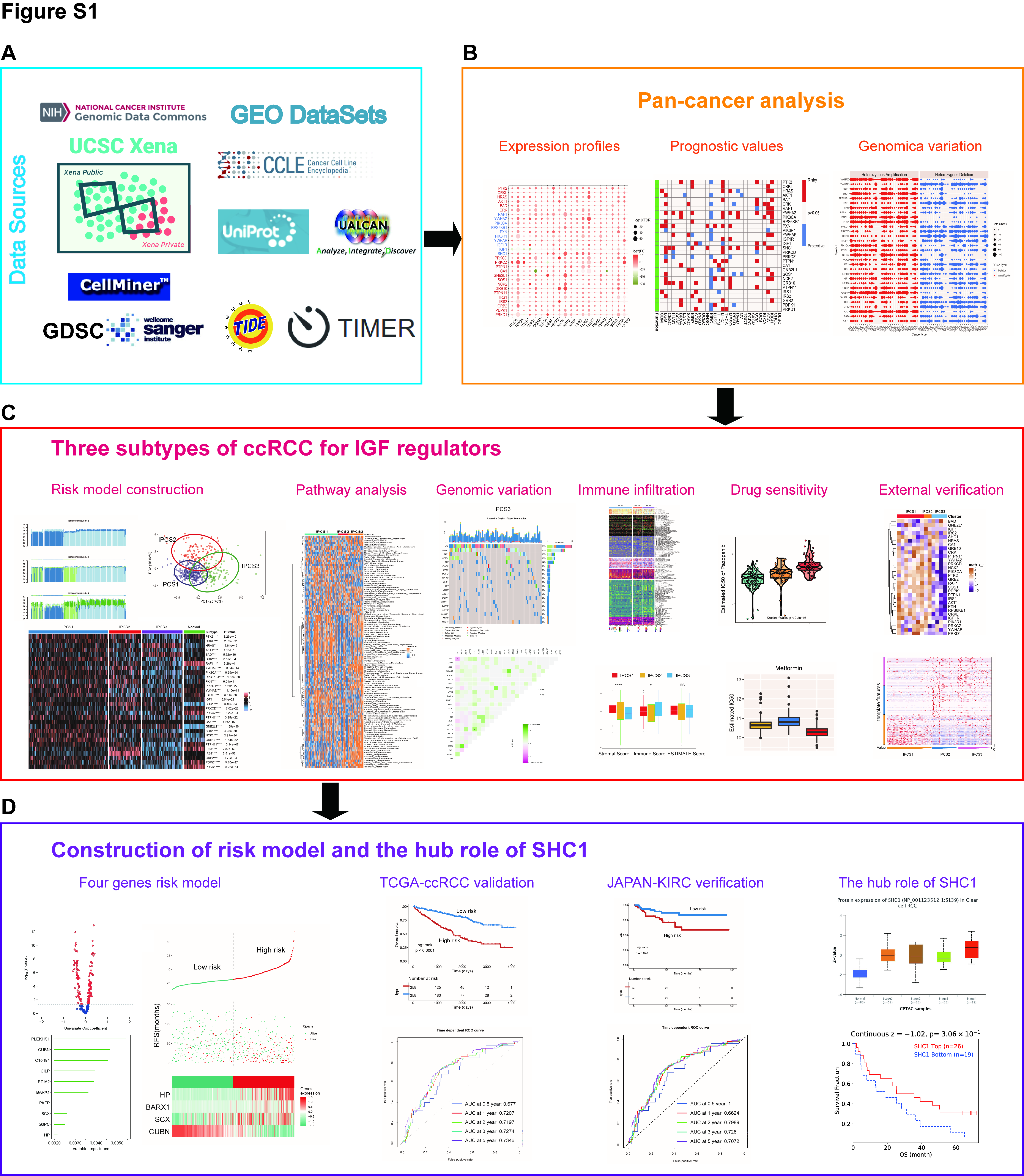

Supplement: Supplementary Figure 1 — Workflow of this study. [file Image_1.tif]

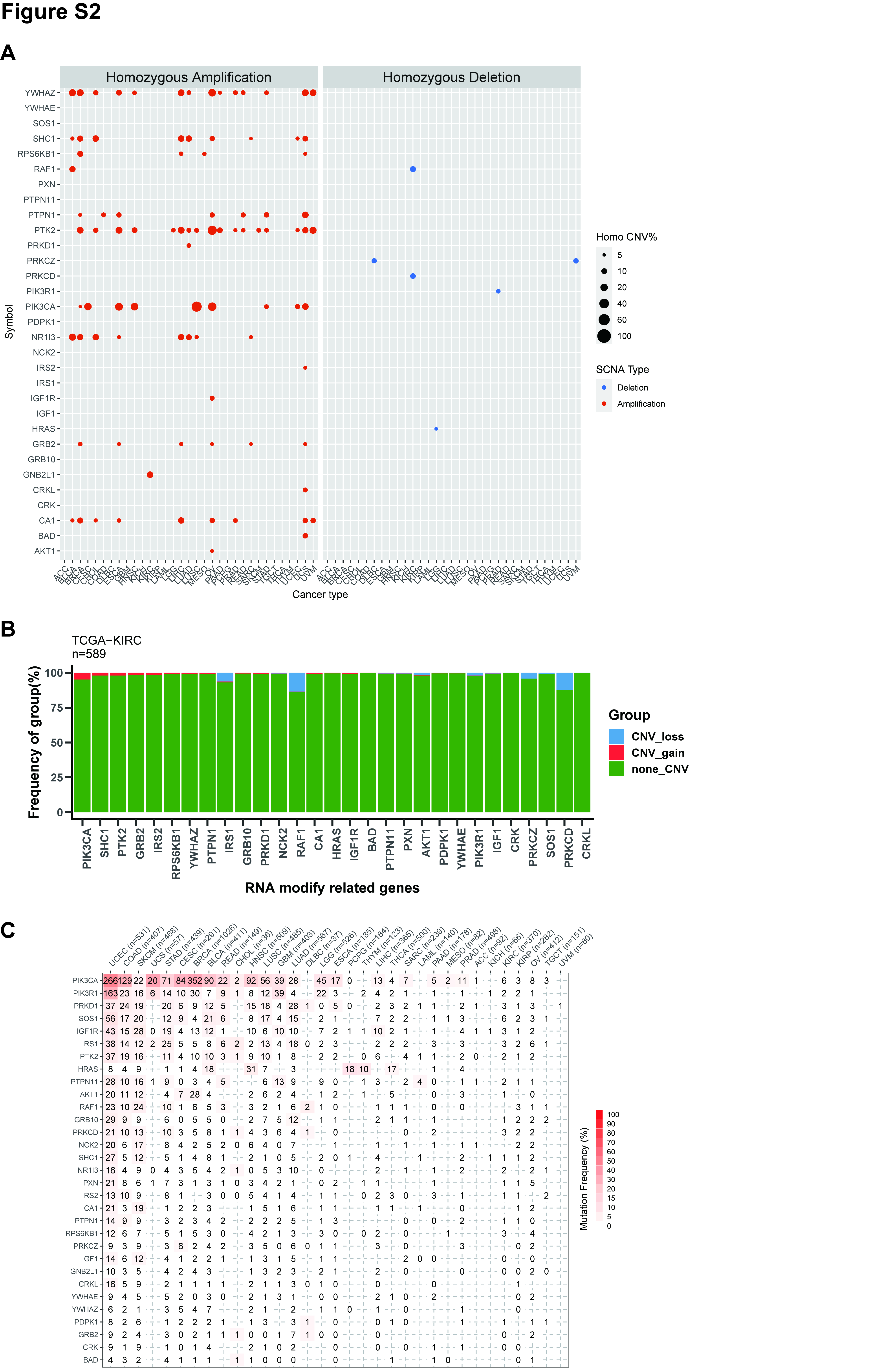

Supplement: Supplementary Figure 2 — Genome mutation profile of IGF regulator genes in multi cancers. (A) The homozygous amplification/deletion of IGF regulator genes. (B) CNV of IGF regulators in TCGA-ccRCC dataset. CNV loss, blue; CNV gain, red; none CNV, green. (C) Mutation frequencies of IGF regulators genes in multi cancers. [file Image_2.tif]

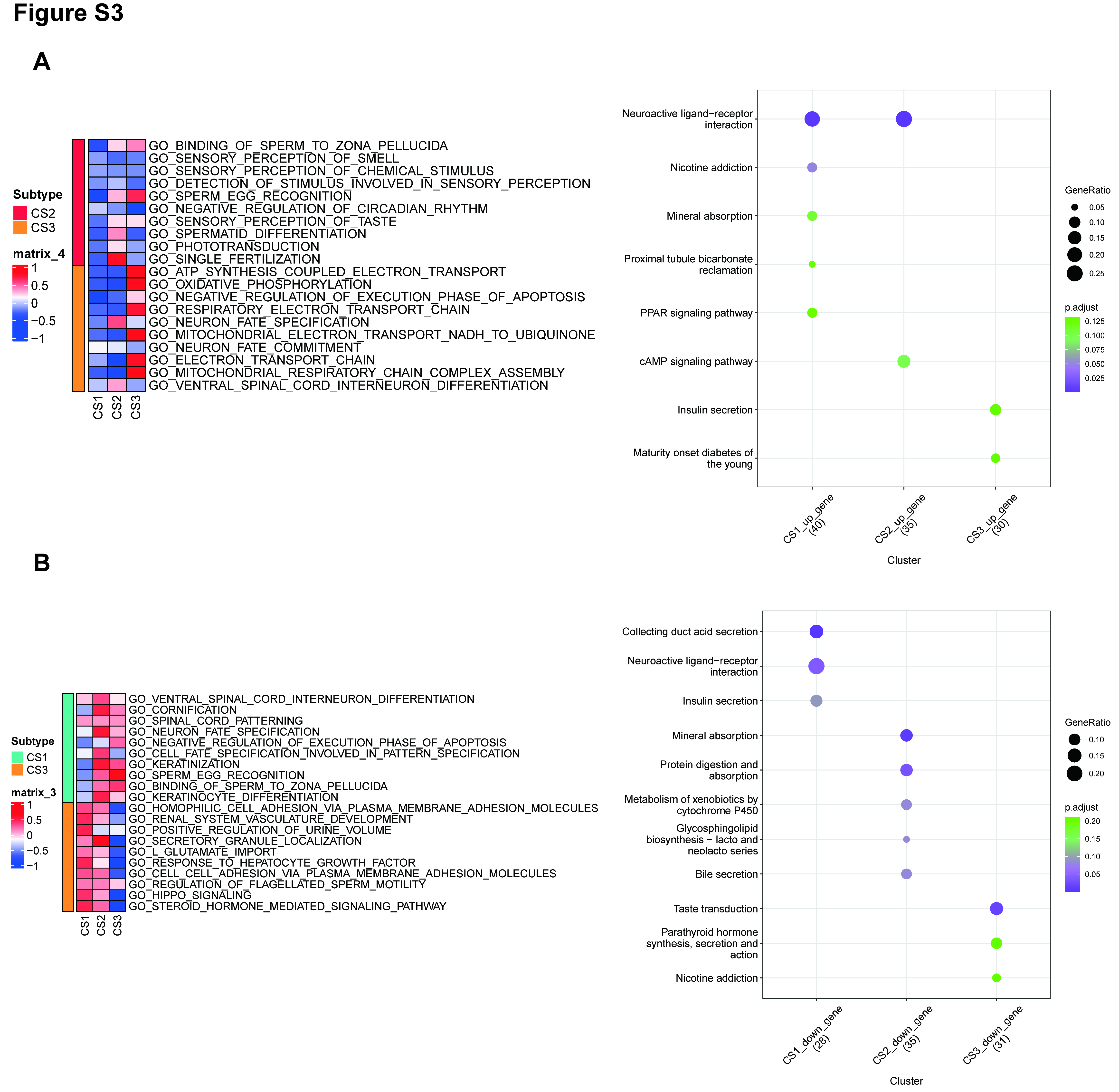

Supplement: Supplementary Figure 3 — Functional enrichment analysis of ccRCC subtypes. (A) GO enrichment analysis of upregulated genes among the three subtypes. (B) GO enrichment analysis of downregulated genes among the three subtypes. [file Image_3.tif]

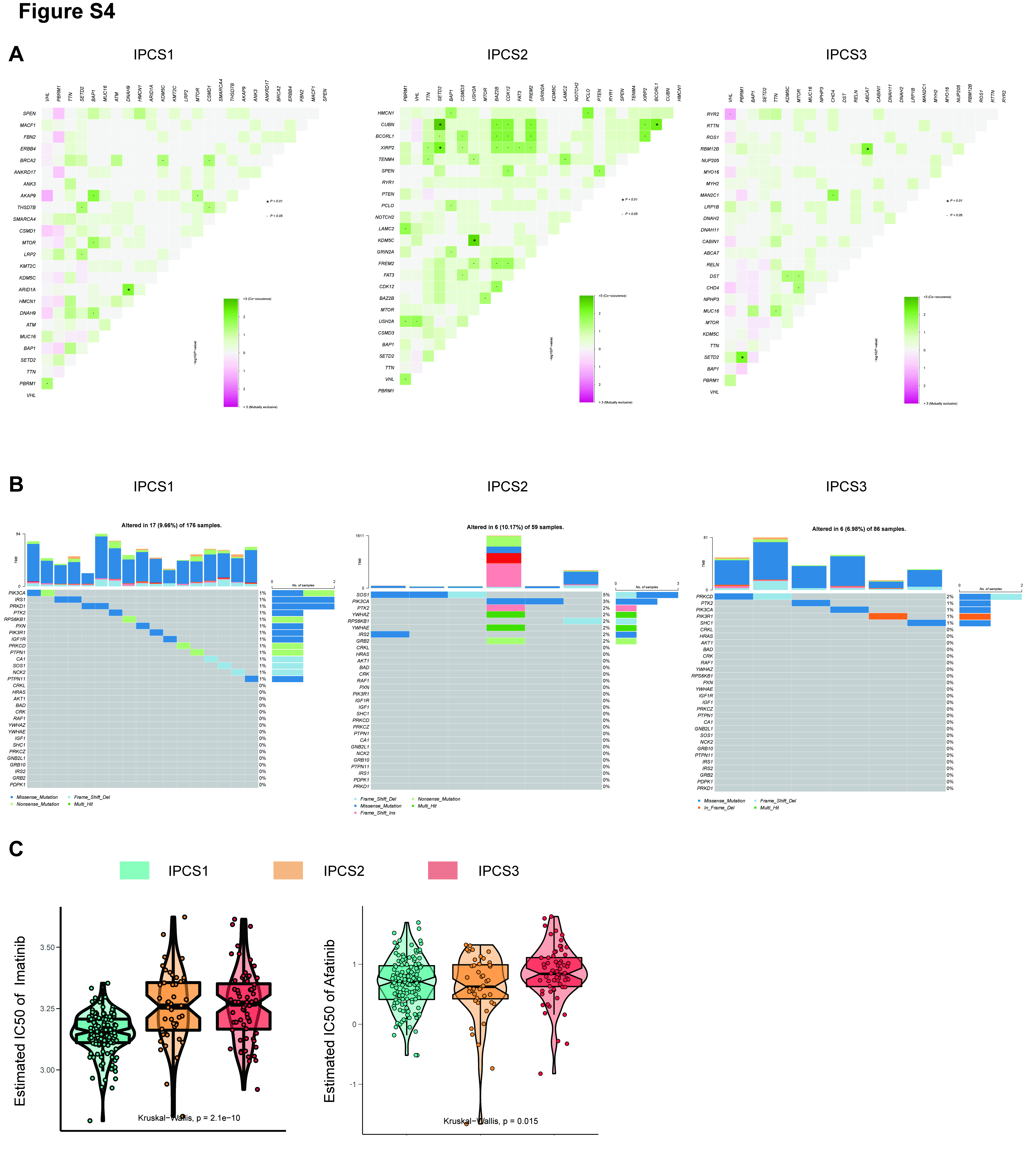

Supplement: Supplementary Figure 4 — The tumor somatic mutations and drug sensitivity assessment among groups. (A) The synthetic lethal mutations in IPCS1, IPCS2 and IPCS3. (B) Waterfall plot indicating the mutation patterns of IGF regulator genes in IPCS1, IPCS2 and IPCS3. (C) Estimated IC50 of the indicated molecular targeted drugs among the three subtypes. [file Image_4.tif]

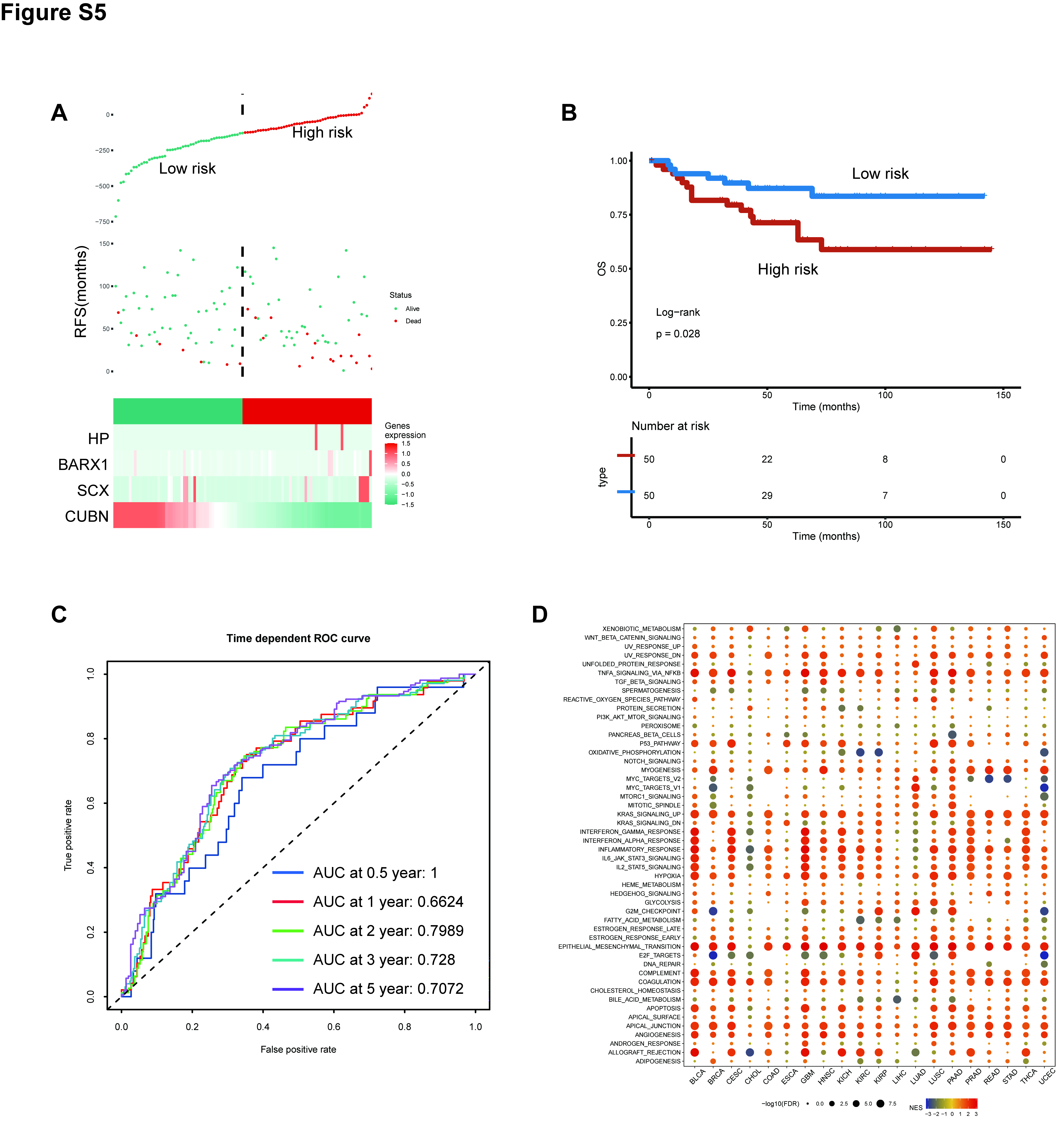

Supplement: Supplementary Figure 5 — Verification of subtyping model in external dataset. (A) Risk score analysis of patients in JAPAN-KIRC cohort. (B) Survival analysis for OS of the two risk signatures in JAPAN-KIRC cohort. (C) The time-dependent ROC curves for the two risk signatures in JAPAN-KIRC cohort. (D) The correlation between SHC1 level and hallmark pathways in KIRC. [file Image_5.tif]
