# Supplementary material for: A New Thinking: Deciphering the Aberrance and Clinical Implication of IGF Axis Regulation Pattern in Clear Cell Renal Cell Carcinoma
Source: Front Immunol. 2022 Jul 22;13:935595. doi: 10.3389/fimmu.2022.935595 (PMC9355597; doi:10.3389/fimmu.2022.935595)
Supplement: Supplementary file 12 [file Table_1.docx]

Summary descriptives table by groups of `group’

|  | **IPCS1** | **IPCS2** | **IPCS3** | **p.overall** |
| --- | --- | --- | --- | --- |
|  | ***N=265*** | ***N=106*** | ***N=135*** |  |
| T: |  |  |  | <0.05 |
| T1 | 157 (59.2%) | 52 (49.1%) | 51 (37.8%) |  |
| T2 | 33 (12.5%) | 12 (11.3%) | 22 (16.3%) |  |
| T3 | 72 (27.2%) | 37 (34.9%) | 59 (43.7%) |  |
| T4 | 3 (1.13%) | 5 (4.72%) | 3 (2.22%) |  |
| N: |  |  |  | 0.206 |
| N1 | 5 (3.50%) | 3 (5.45%) | 7 (8.75%) |  |
| NX | 138 (96.5%) | 52 (94.5%) | 73 (91.2%) |  |
| M: |  |  |  | 0.306 |
| M1 | 27 (77.1%) | 21 (84.0%) | 27 (67.5%) |  |
| MX | 8 (22.9%) | 4 (16.0%) | 13 (32.5%) |  |
| grade: |  |  |  | . |
| G1 | 9 (3.40%) | 2 (1.89%) | 1 (0.74%) |  |
| G2 | 135 (50.9%) | 40 (37.7%) | 42 (31.1%) |  |
| G3 | 99 (37.4%) | 40 (37.7%) | 62 (45.9%) |  |
| G4 | 21 (7.92%) | 21 (19.8%) | 29 (21.5%) |  |
| GX | 1 (0.38%) | 3 (2.83%) | 1 (0.74%) |  |
| stage: |  |  |  | 0.001 |
| i | 154 (58.1%) | 51 (48.1%) | 49 (36.3%) |  |
| ii | 30 (11.3%) | 11 (10.4%) | 14 (10.4%) |  |
| iii | 53 (20.0%) | 22 (20.8%) | 42 (31.1%) |  |
| iv | 28 (10.6%) | 22 (20.8%) | 30 (22.2%) |  |
| sex: |  |  |  | 0.009 |
| female | 104 (39.2%) | 24 (22.6%) | 45 (33.3%) |  |
| male | 161 (60.8%) | 82 (77.4%) | 90 (66.7%) |  |
| age | 59.8 (12.4) | 60.9 (11.3) | 61.1 (12.6) | 0.502 |
| OS: |  |  |  | 0.001 |
| 0 | 197 (74.3%) | 63 (59.4%) | 77 (57.0%) |  |
| 1 | 68 (25.7%) | 43 (40.6%) | 58 (43.0%) |  |
| OS.time | 1449 (969) | 1361 (933) | 1354 (1015) | 0.569 |
| PFI: |  |  |  | <0.001 |
| 0 | 212 (80.0%) | 59 (55.7%) | 78 (57.8%) |  |
| 1 | 53 (20.0%) | 47 (44.3%) | 57 (42.2%) |  |
| PFI.time | 1301 (957) | 1081 (901) | 1132 (930) | 0.067 |
